# Brighter Days May Be Ahead: Continuous Measurement of Pediatric Intensive Care Unit Light and Sound

**DOI:** 10.3389/fped.2020.590715

**Published:** 2020-10-26

**Authors:** Kara D. Greenfield, Oliver Karam, A. M. Iqbal O'Meara

**Affiliations:** Children's Hospital of Richmond at Virginia Commonwealth University, Richmond, VA, United States

**Keywords:** light, sound, PICU, delirium, intensive care, hospital environment

## Abstract

**Objective:** To describe light and sound characteristics in the rooms of critically ill children.

**Design:** Prospective observational cohort study, with continuously measured light and sound levels.

**Setting:** Tertiary care pediatric intensive care unit (PICU), with a newly constructed expansion and an older, pre-existing section.

**Patients:** Critically ill patients 0–18 years old, requiring respiratory or cardiovascular support. Patients with severe cognitive pre-conditions were excluded.

**Measurements and Main Results:** One hundred patients were enrolled, totaling 602 patient-days. The twenty-four hour median illuminance was 16 (IQR 5-53) lux (lx). Daytime (07:00–21:00) median light level was 27 lx (IQR 13-82), compared with 4 lx (IQR 1-10) overnight (22:00–06:00). Peak light levels occurred midday between 11:00 and 14:00, with a median of 48 lx (IQR 24-119). Daytime median illuminance trended higher over the course of admission, whereas light levels overnight were consistent. Midday light levels were higher in newly constructed rooms: 78 lx (IQR 30-143) vs. 26 lx (IQR 20-40) in existing rooms. The twenty-four hour median equivalent sound level (LAeq) was 60 (IQR 55-64) decibels (dB). Median daytime LAeq was 62 dB (IQR 58-65) and 56 dB (IQR 52-61) overnight. On average, 35% of patients experienced at least one sound peak >80 dB every hour from 22:00 to 06:00. Overnight peaks, but not median sound levels nor daytime peaks, decreased over the course of admission. There was no difference in sound between new and pre-existing rooms.

**Conclusions:** This study describes continuously measured light and sound in PICU rooms. Light levels were low even during daytime hours, while sound levels were consistently higher than World Health Organization hospital room recommendations of <35 dB. Given the relevance of light and sound to sleep/wake patterns, and evidence of post-intensive care syndromes, the clinical effects of light and sound on critically ill children should be further explored as potentially modifiable environmental factors.

## Introduction

Though intended as places of healing, Pediatric Intensive Care Unit (PICU) environments are often a significant source of stress for patients. Around the clock medical interventions, machine and alarm noise, and nighttime light not only contribute to this stress, but prevent restorative sleep at a time of physiologic vulnerability. Severity of illness, inactivity, mechanical ventilation, and exposure to sedatives and other drugs have also been associated with disrupted sleep in the adult intensive care unit (ICU). These disruptions include decreased sleep quantity and quality, increased daytime sleep, and disrupted sleep architecture, as well as altered melatonin synthesis and circadian rhythm disturbance, which may affect hormonal regulation, cellular and organ function, and ultimately, patient outcomes (1, 2). Studies using polysomnography (PSG) in both adult and pediatric ICUs show a significant reduction or absence of deeper, slow wave sleep and rapid eye movement (REM) sleep, with an increase in nighttime arousals and daytime sleep (1–5). As much as 60% of total sleep time in the ICU is spent in stage 1, which is the lightest, and typically most transient, phase (5). Sleep cycle duration varies with age, though normally <5% of total sleep time is spent in stage 1 (1, 2). Adult studies have linked sleep and environmental disruptions with the development of delirium, and it stands to reason that the same is true for critically ill children (6–8). Delirium is increasingly recognized in children, and is independently associated with increased length of stay and ventilator days, neurologic morbidity, and mortality (9, 10).

The World Health Organization (WHO) recommends hospital room noise not exceed 35 dB (11). Existing literature suggests that these guidelines are rarely met (3, 12–15). As such, previous work has linked hospital noise with disruption of patient sleep, increased need for sedation, increased length of stay, and patient stress (14, 16–18). There are no similar WHO recommendations for hospital lighting, and frequent nighttime, and low daytime, light exposures have been observed in intensive care environments (19). Little PICU-specific data regarding sound or light patterns exists, as the majority of existing literature encompasses brief (<24–48 h) periods of data collection in adult units. Frequent and sometimes constant presence of parents or guardians, and even young siblings, is an aspect of the PICU environment that may differ from adult units.

Implementation of multicomponent bundles has been found to be an effective strategy in reducing delirium and improving survival, and these bundles commonly include light- and sound-based interventions (opening blinds during daytime hours, imposing unit-wide 'quiet time', clustering of care, etc.) (8, 20, 21). Recently, pediatric intensive care early mobility and rehabilitation initiatives such as “PICU Up!” also show promise for improving functional survival (22). Interestingly, the majority of these types of studies have not reported baseline light and sound characteristics, nor have they demonstrated whether bundle or mobility interventions inherently alter environmental characteristics, and if this aspect is uniquely beneficial. If found to be therapeutic, environmental interventions might be independently useful adjuncts in patients less able to mobilize. The purpose of this study was to describe longitudinal trends of light and sound in the rooms of critically ill children during PICU admission. A secondary objective was to identify differences between new construction PICU rooms built to the 2010 Guidelines for Design and Construction of Health Care Facilities, and older, pre-existing rooms constructed in 1993 before new regulations (23).

## Materials and Methods

### Study Design

This is a prospective observational single-center study involving children admitted to the PICU of the Children's Hospital of Richmond. This study was reviewed and approved with a waiver of informed consent by the Institutional Review Board of Virginia Commonwealth University.

### Study Population

In preparation for a study of PICU environment and delirium interventions, we conducted a prospective observational cohort study of 100 consecutive critically ill patients in an academic, tertiary care PICU from August 2018 through March 2019 to establish an environmental baseline. Data was collected from PICU admission through PICU day 14 or transfer or discharge from the PICU, whichever came first. Children ≤18 years were eligible for the study if they were admitted to a room with light and sound meters for more than 24 h. Patients were included if they were critically ill, defined as requiring invasive or non-invasive positive pressure ventilation or vasoactive medication during their stay. Severity of illness at admission was measured by the PELOD-2 score (24). Patients with severe developmental delay, defined as Pediatric Cerebral Performance Category (PCPC) >4 at pre-hospital baseline, were excluded, as the Cornell Assessment of Pediatric Delirium (CAPD) has poor specificity in that population (25, 26).

### Data Collection

**Figure 1:**
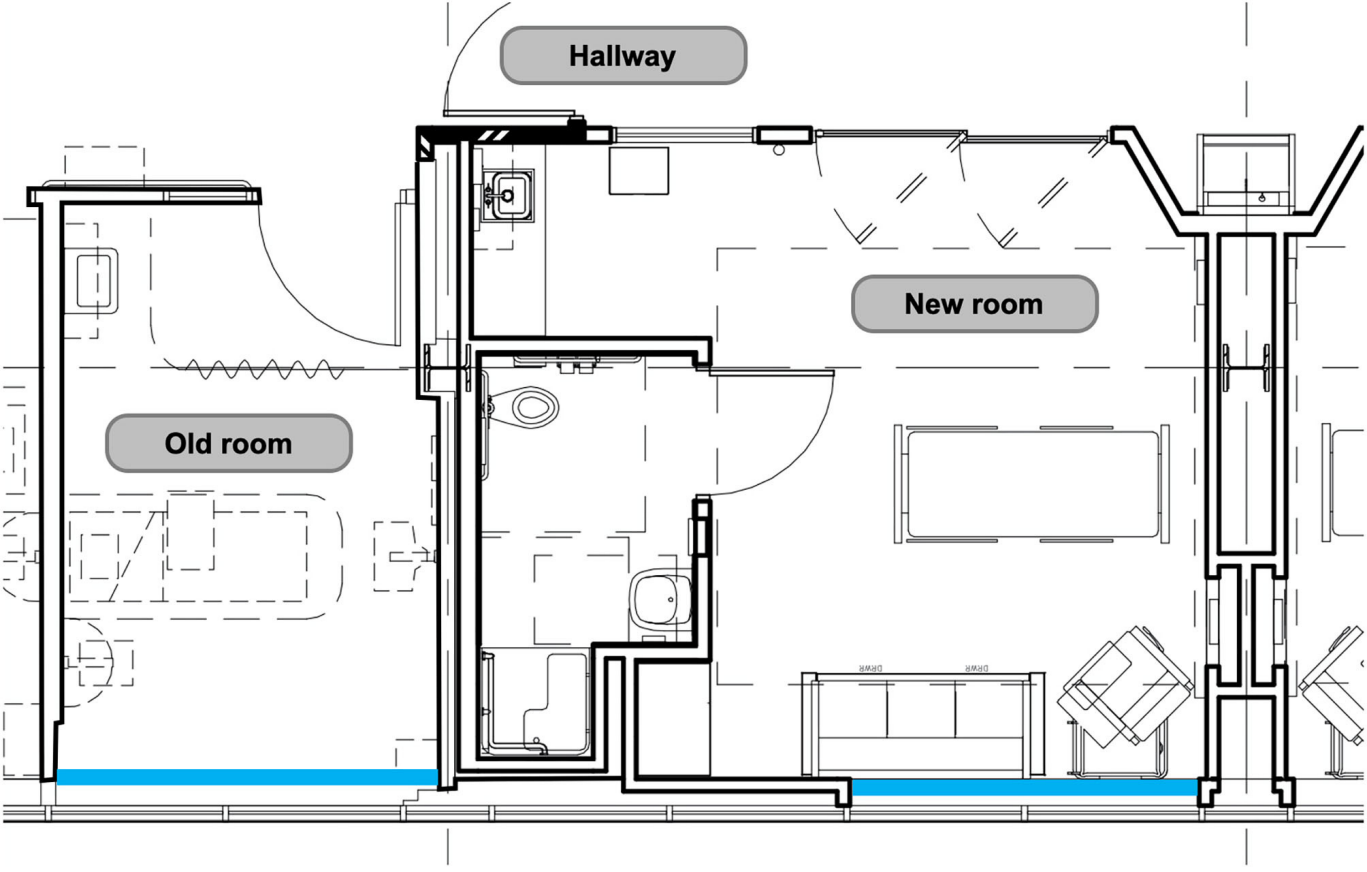
Blueprint of existing and new construction PICU rooms. Windows are outlined in blue.

As the number of rooms with meters placed was limited to rooms located along the same hallway, with south-facing windows, light and sound data were continuously measured in thirteen individual patient rooms out of our twenty beds. Recent unit expansion allowed for the placement of meters in six newly constructed rooms (built in 2017) and seven existing rooms (built in 1993). In comparison to the existing rooms, newly constructed rooms are larger in size, with more overhead lighting and lighting options, have proportionally larger windows and glass sliding doors, and are painted a brighter color (Figure 1). Light and sound meters were mounted above the head of bed; this location was chosen in an effort to best simulate the patient experience while minimizing impact on patient care.

This study used Reed Instruments light meters (SD-1128, Reed Instruments, Wilmington, NC, USA) and sound level meters (SD-4023, Reed Instruments, Wilmington, NC, USA). The devices were calibrated prior to installation according to manufacturer recommendations. Meters were equipped with secure digital (SD) memory cards for data storage. Light meters measured light in lux, with a sampling rate of 30 s. When referencing visible light, the typical measurement unit is lux (lx). Many building guidelines suggest classrooms and offices be 300–500 lx, hallways and stairwells 50–100 lx (27). Bright, direct sunlight is more than 100,000 lx, overcast daylight about 1,000 lx, and a night with a full moon <1 lx. Sound meters measured sound in decibels (dB) at a sampling rate of 2 s on a fast, A-weighted scale. The decibel is a logarithmic scale, and a validated measure of the human perception of sound pressure intensity. The average person can hear sounds as low as 0 dB, while the pain threshold is near 140 dB. A change of 3–5 dB can be perceived by the human ear, and a change of 10 dB is perceived as a doubling of volume (28). For reference, a whisper is about 30 dB, with normal conversation occurring at 60 dB; a library is ~40 dB, garbage disposal 80–95 dB, and a leaf blower 110 dB (29).

### Analysis

Monitor data were collected in Microsoft Excel and uploaded to R program (R Foundation for Statistical Computing, Vienna, Austria) for analysis. Light levels were averaged in 30 min intervals, with maximum and minimum values taken every hour. Equivalent sound was calculated at 15 min intervals, with hourly minimum and maximum measurements. Sound peaks, defined as the number of times a room's sound level was above various thresholds, from 70 to 90 dB, were also collected. Patient demographics were extracted from the electronic medical record and entered in a Research Electronic Data Capture (REDCap) database, hosted at Virginia Commonwealth University.

Demographic characteristics between the new vs. pre-existing unit were compiled, and chi-square tests were performed for these categorical values. Light and sound data are presented in a descriptive manner, without tests to assess statistical significance. With more than a million data points, even a minor difference in light or sound would likely be statistically significant. Results are presented as number and percentage or median and interquartile range (IQR), as appropriate. Additionally, results are presented as “daytime” (07:00–21:00) or “nighttime” (22:00–06:00). These times were chosen with the concept of merging a typical bedtime with the observed natural bustle of the ICU at shift changes. Light and sound levels between the new construction and existing PICU rooms were compared. Light and sound level daily trends over the PICU stay were also evaluated.

## Results

### Patient Characteristics

One hundred patients were analyzed, totaling 602 patient days. A total of 336 patients were admitted to rooms with light and sound meters during the study period. Eighty four were excluded for length of stay <24 h, 10 for light and/or sound meter malfunction, and 142 patients did not meet illness severity or neurologic criteria. As shown in Table 1, 62% of patients were male, with median age 18 months (IQR 3-71). Median length of stay was 4 days (IQR 3-8). Forty seven percentage of patients were African American, 28% white, 13% Hispanic or Latino. Median severity of illness at admission, as indicated by the PELOD-2 score, was 2 (IQR 0-5). Cardiac patients were only admitted to the newly constructed portion of the unit.

**Table 1:** Patient characteristics.

**Patient characteristic**	**Combined units (*n* = 100)**	**New unit (*n* = 69)**	**Old unit (*n* = 31)**	***p*-value**
Age (mo), median (IQR)	18 (3–77)	19 (4–109.5)	13 (2–51)	0.2
Weight (kg), median (IQR)	10 (5.9–22.1)	10.5 (6.1–30.8)	9.9 (5.3–15.9)	0.17
Sex (male), *n* (%)	62 (62%)	41 (59%)	21 (68%)	0.4
Diagnosis at admission *some patients have multiple diagnoses				
Respiratory	68 (68%)	46 (67%)	22 (71%)	0.67
Cardiac	10 (10%)	10 (15%)	0 (0%)	**0.026**
Neurologic	7 (7%)	3 (4%)	4 (13%)	0.123
Trauma	8 (8%)	5 (7%)	3 (10%)	0.68
Post-operative	22 (22%)	16 (23%)	6 (19%)	0.67
Infectious	47 (47%)	30 (44%)	17 (55%)	0.295
Oncologic	2 (2%)	2 (3%)	0 (0%)	0.341
Other	5 (5%)	4 (6%)	1 (3%)	0.587
Ethnicity				0.049
White	28 (28%)	23 (33%)	5 (16%)	
African American	47 (47%)	31 (45%)	16 (52%)	
Hispanic	13 (13%)	10 (15%)	3 (10%)	
Other	12 (12%)	5 (7%)	7 (23%)	
PELOD-2	2 (0–5)	2 (0–6)	2 (0–5)	0.51

*The bold value indicates p-value < 0.05*.

### Light

**Figure 2:**
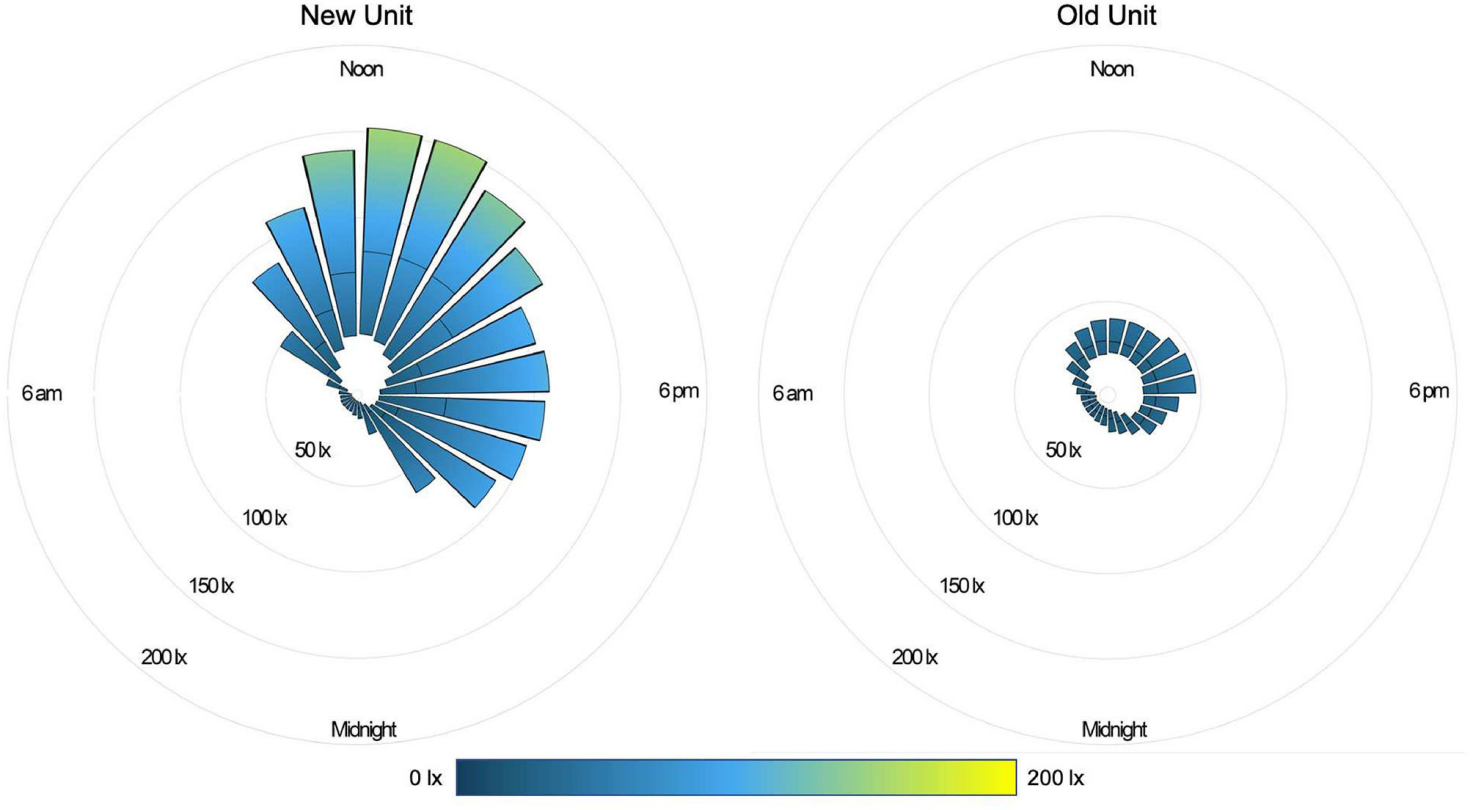
Light in the PICU: 24 h illuminance (median, IQR) for all patient days by location.

**Figure 3:**
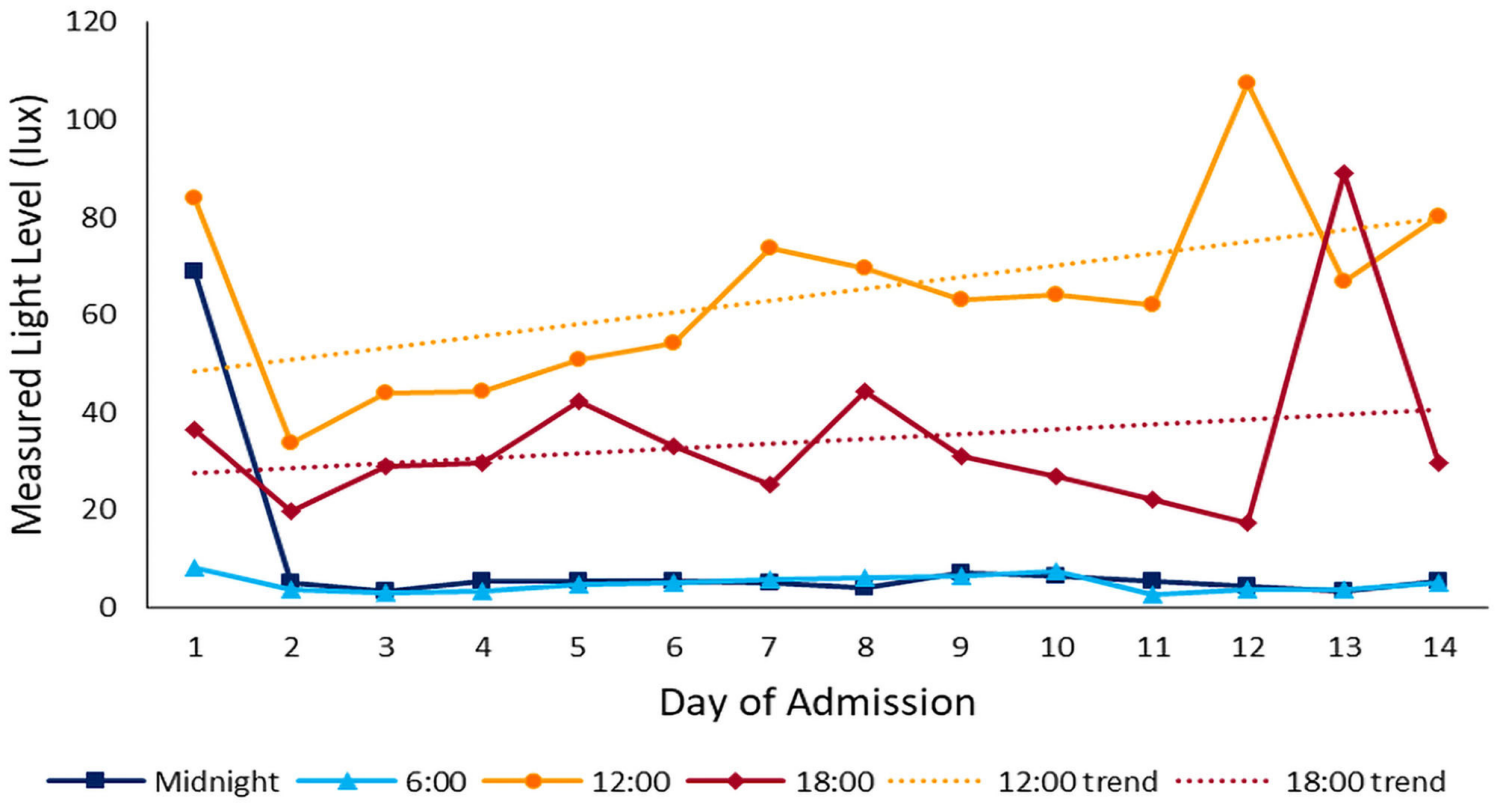
Light throughout admission: median light levels at 4 different time points throughout the day over the course of admission. Daytime light levels increase over the course of admission.

During the daytime (07:00–21:00), the median light level was 27 lx (IQR 13-82), compared with 4 lx (IQR 1-10) overnight (22:00–06:00). Peak illuminance in all PICU rooms was reached during midday hours (11:00–14:00); median light level during these hours was 48 lx (IQR 24-119). Figure 2 shows median and IQR light levels over the course of the day for all patient days by new and old units. New rooms, with proportionately larger windows and improved lighting, were measurably brighter. The median daytime illuminance was 38 lx (IQR 12-107) in new rooms compared with 23 lx (IQR 15-34) in older rooms. During the typically brighter midday hours, new rooms were much brighter, with a median of 78 lx (IQR 30-143) while older rooms were 26 lx (IQR 20-40). All rooms, regardless of construction date, were darker at night. New rooms had a median of 3 lx (IQR 0–8) during overnight hours and existing rooms 8 lx (IQR 3-13). Figure 3 depicts median light levels in both units by day of admission at four time points throughout the day. Overnight light levels (midnight, 0600) remained low on each day of admission; rooms were comparatively brighter during daytime hours (noon, 1800), which increased over the course of admission.

### Sound

**Figure 4:**
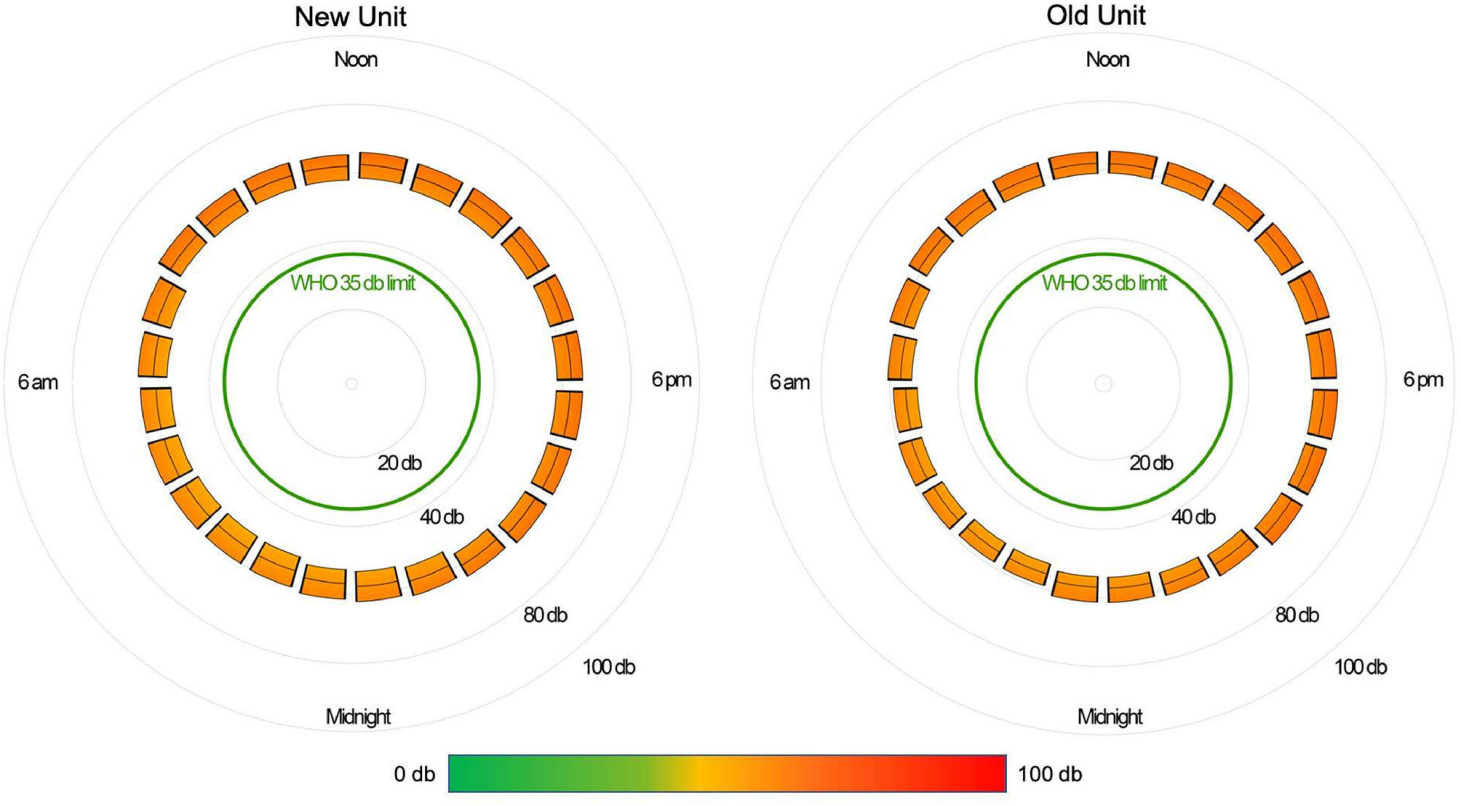
Sound in the PICU: 24 h sound levels (median, IQR) by location.

**Figure 5:**
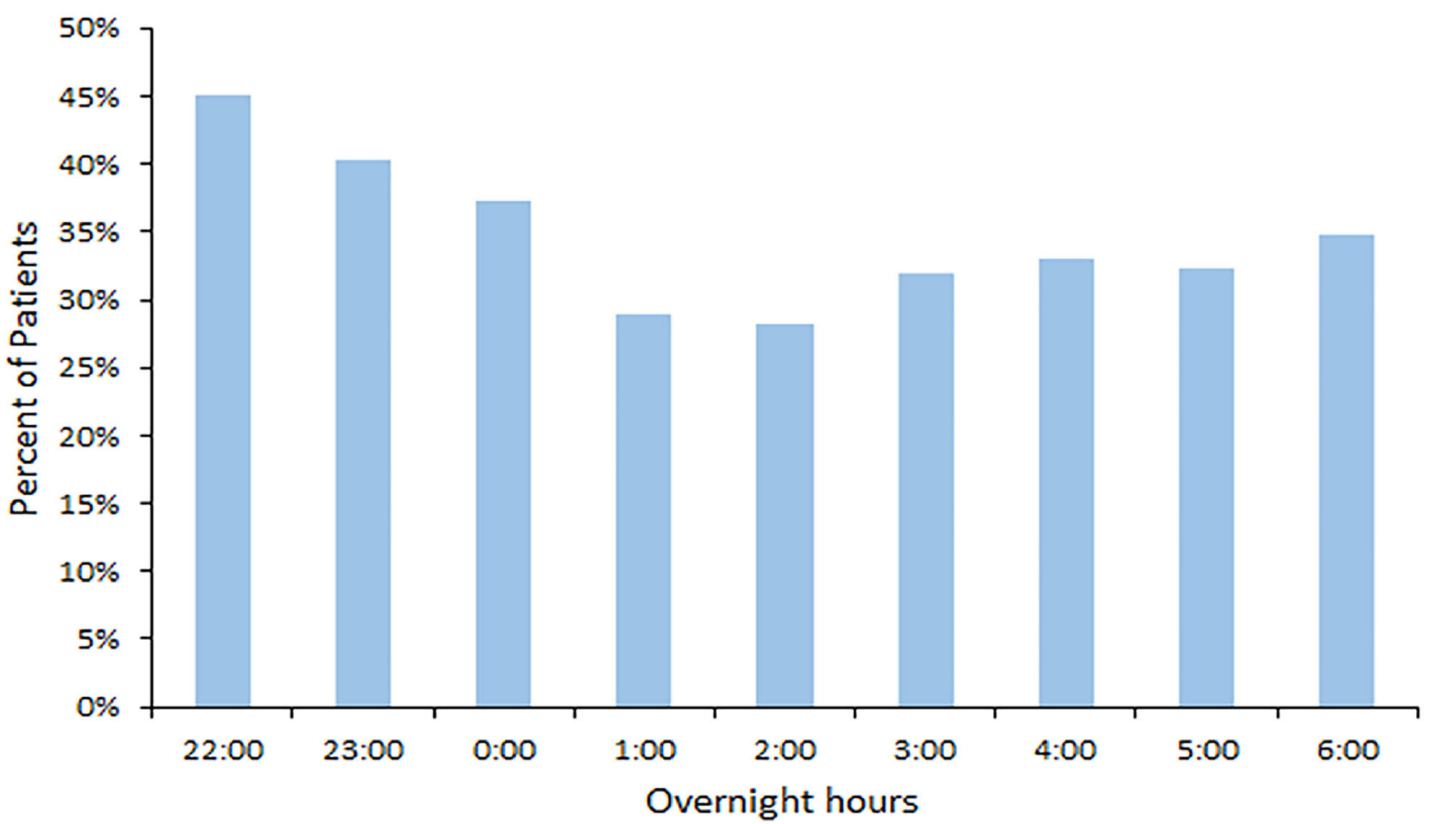
Overnight Sound Peaks: percentage of patients experiencing sound peaks >80 dB each hour from 22:00 to 06:00.

**Figure 6:**
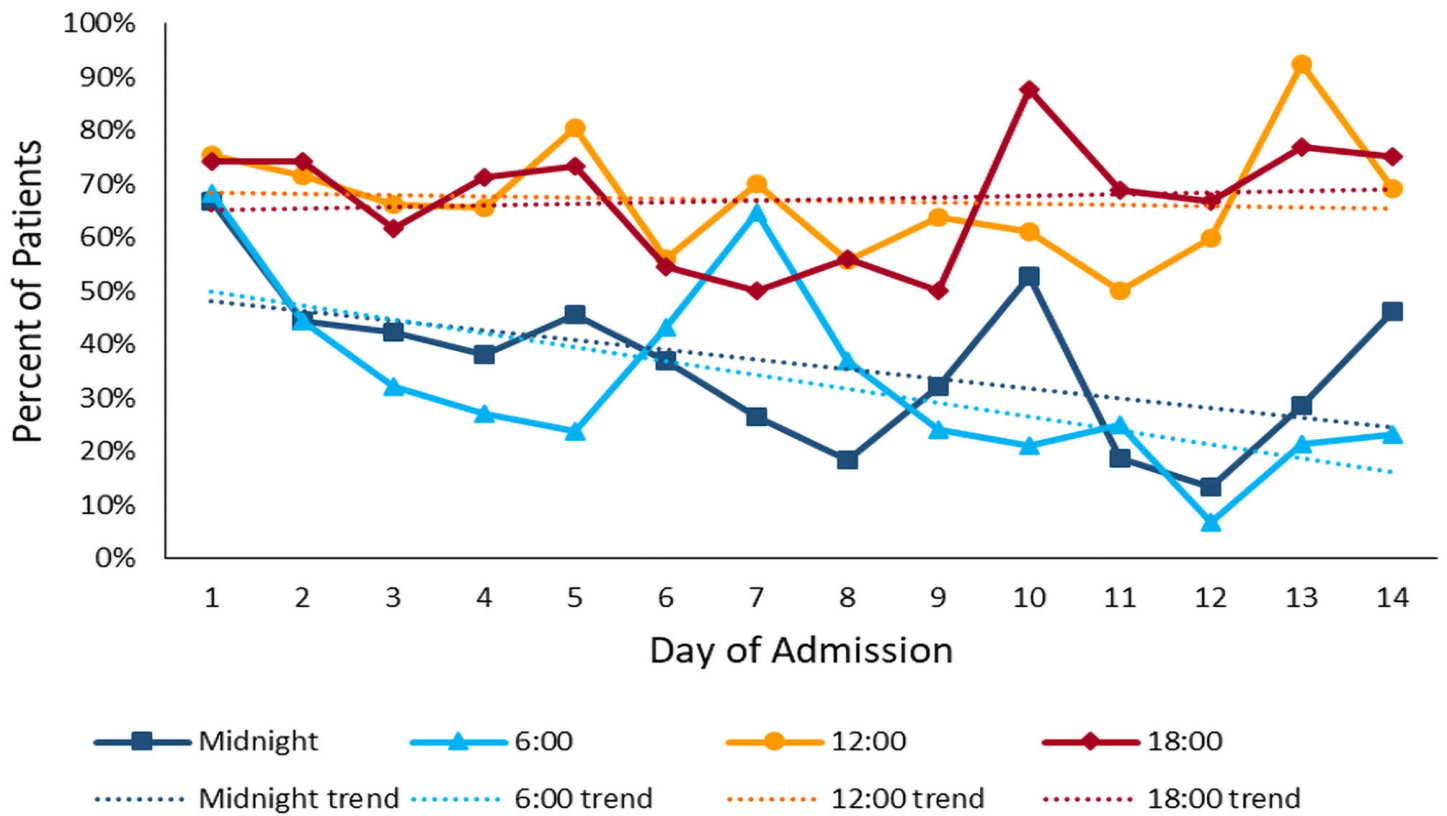
Sound Peaks throughout admission: percentage of patients experiencing sound peaks >80 dB at four time points throughout the day over the course of admission. Overnight sound peaks trend downward over the course of admission.

The median daytime equivalent sound level for all patient days was 62 dB (IQR 58-65), compared with 56 dB (IQR 52-61) overnight. Sound levels were nearly identical between new and existing PICU rooms; Figure 4 depicts median and IQR sound levels for new and existing units for all patient days. New construction PICU rooms were 62 dB (IQR 57-65) during daytime hours and 56 dB (IQR 52-61) overnight, compared with existing rooms 61 dB (IQR 58-65) during the daytime and 56 dB (IQR 53-60) overnight. Data collected from an empty room indicate the lowest sound level achieved was 46 dB. Sound peaks were frequent and occurred at all times of the day and night. The number of patients experiencing sound peaks was similar between the two units. Figure 5 shows the percentage of patients experiencing sound peaks >80 dB during overnight hours in all PICU rooms. On average, 35% of patients experienced peaks >80 dB during these hours. Sound peak height did demonstrate a day/night pattern, with an average of 62% of patients experiencing daytime peaks >80 dB each hour between 07:00 and 21:00 (daytime peak sound data not shown). Figure 6 illustrates the percentage of patients experiencing sound peaks >80 dB by day of admission at four time points throughout the day. The number of patients with overnight sound peaks down-trended by day of admission.

## Discussion

Although several studies have addressed noise levels in pediatric intensive care units, there is little evidence of combined assessment of light and sound levels over a period of time (3, 13, 14, 30–32). This study is unique, as it looks at continuous trends in both the light and sound environments of pediatric patient rooms throughout their stay. We found a small day/night difference in light levels in our unit. However, daytime light levels are remarkably low; they do not mimic natural daylight, nor do they approximate commonly accepted building standards which are at least several hundred lux during daytime hours. With respect to sound, our unit has little variability between daytime and nighttime levels. As the decibel scale is logarithmic, median sound is at least four times louder than the WHO-recommended hospital sound levels at all hours of the day. Also striking are the number of sound peaks occurring each hour, particularly during overnight hours.

There is little data on longitudinal light trends in adult or pediatric intensive care units; published studies demonstrate variability across ICUs. Meyer et al. measured light for seven days across multiple units. In a three-bed ICU room, there was clear diurnal variation, with daytime light reaching 1000 lx and nighttime light levels near 0 lx (12). A multi institutional study of seven ICUs in China measured light and sound levels over a 24 h period and found the majority had levels <200 lx at all hours, when measured at patient eye level; many of the units also showed little diurnal variation (15). Our findings were similar, with a small difference between median daytime and nighttime light levels: 35 lx in the newly constructed rooms and 15 lx in existing rooms. Despite newly constructed rooms being larger in size, with more overhead lighting, proportionally larger windows, and brighter wall color, these rooms never reached the 250 lx of a typical shady room in daylight for any period sufficient to impact the every 30 min light average. They certainly were numerically brighter, which may reflect interval changes in hospital construction standards. Interestingly, our unit implemented delirium screening within a year prior to this study, with prompting for non-pharmacologic interventions to prevent and treat delirium, including implementation of a day/night cycle; however, there is no unit policy regarding timed light cycling.

Similar to nearly all prior studies where ICU sound was measured, our results demonstrate that sound levels in our unit are consistently higher than the WHO recommendations of <35 dB. Our study differs from prior studies in that we continuously measured sound throughout patients' ICU stays. As such, we effectively controlled for day to day variability in staff, and patient- and unit-specific activity. Despite this, similar to prior studies, our rooms showed minimal day/night variation (3, 12–15, 32). Additionally, despite substantial differences in size and construction materials, both newly constructed and existing rooms had nearly identical sound levels.

The higher-than-recommended median sound levels are notable, though perhaps more clinically relevant are the number of sound peaks, particularly during overnight hours. Sound peaks reflect a disruption in the average sound perceived by the patient. This may make uninterrupted, quality sleep more difficult to achieve. Imagine falling asleep on an airplane, where the noise level is consistently high, in comparison to falling asleep in a classroom with someone intermittently clapping his hands in your vicinity. More than 80% of patients in our study experienced at least one sound peak >70 dB every hour. With ambient nighttime sound levels of 56 dB, a sound suddenly >70 dB would be perceived as more than a doubling of loudness. A study in a pediatric cardiac ICU found that high equivalent sound levels were associated with increased sedation requirements, and high hourly maximum sound levels were associated with need for additional sedation within 2 h (14). Given the association of sedation factors with delirium and poor neurologic outcomes, identifying easily modifiable factors could facilitate functional survivorship, as well as patient and family satisfaction.

Our PICU is dark and loud. Modifying day/night lighting to promote circadian physiology is feasible, and there are newly available technologies to remotely control unit lighting and to alter lighting temperature by time of day. However, there are no established best practice parameters for the critically ill aside from unspecified increase of daytime light and activity alternated with nighttime darkness. It is challenging to alter the background noise of necessary patient care equipment such as mechanical ventilators and extracorporeal support circuits. However, with evidence-based, targeted interventions, provider education, and culture change, it may be possible to reduce the number of jarring sound peaks, particularly in the overnight hours. Other possible interventions include earplugs or noise-canceling headphones, noise-reducing floor and ceiling tiles, acoustic wall covering or paint, and auto-programmed volume reduction of patient monitors and infusion pumps. Daytime inclusion of familiar or orienting sounds such as music or a parent's voice may also have a role.

There are several limitations of this study, including the single-center setting. Not all PICU rooms were equipped with light and sound monitoring, as some are north-facing. It is possible that patients in the north-facing rooms were different, although we do not have a policy influencing where patients are placed. All rooms with enrolled patients had south-facing windows, though there is daylight variability given that the hospital is situated on a city block surrounded by buildings that alter the natural light in some rooms, and patients were admitted during multiple seasons. Patients who did not require respiratory or circulatory support, as well as patients with severe developmental delay were excluded from the study. As such, this sample is not representative of all-comers to the PICU. Recent validation of CAPD delirium scoring in developmentally delayed children will allow for their inclusion in future studies (33). Additionally, light and sound meter placements were chosen, in part, to minimize interruptions to patient care, and the data may not represent the exact patient experience if the room setup differed based on patient care needs. We were not able to measure exposures to very direct light, such as with the assessment of pupillary reflexes. Nor were we able to account for the ambient noise of respiratory support transmitted directly to the patient, or identify the cause of sound peaks, as there was no correlative observation of patients' surroundings. Finally, as an observational study of our unit environment, we did not evaluate illness severity, diagnosis, or specific therapies and their relation to light or sound.

## Conclusions

This study describes the longitudinal trends of continuously measured light and sound during PICU admission in our institution. We report a surprisingly low daytime light level. There was little day/night difference in sound levels. Notably, a majority of patients experienced marked sound peaks overnight. While our unit may not represent every PICU, this study calls attention to the need for environmental awareness in pediatric critical care medicine. Future studies are needed to determine the clinical significance of light and sound, including correlation with, and effect on, illness severity, development of delirium or other acquired neurologic morbidity, need for sedatives/analgesics, length of stay, and mortality. Simultaneous electroencephalography or measurements of melatonin or vitamin D may also be informative in developing evidence-based environmental therapeutic strategies.

## Data Availability Statement

The raw data supporting the conclusions of this article will be made available by the authors, without undue reservation, to any qualified researcher.

## Ethics Statement

The studies involving human participants were reviewed and approved by Virginia Commonwealth University Institutional Review Board. Written informed consent from the participants' legal guardian/next of kin was not required to participate in this study in accordance with the national legislation and the institutional requirements.

## Author Contributions

KG wrote the research project, analyzed data, and wrote the manuscript. OK wrote the research project, analyzed the data, and reviewed the manuscript. AI wrote the research project and reviewed the manuscript. KG and OK had full access to the data and take responsibility for its integrity. All authors contributed to the article and approved the submitted version.

## Conflict of Interest

The authors declare that the research was conducted in the absence of any commercial or financial relationships that could be construed as a potential conflict of interest.
